# Identification of C-terminal hydrophobic residues important for dimerization and all known functions of ParB of *Pseudomonas aeruginosa*

**DOI:** 10.1099/mic.0.056234-0

**Published:** 2012-05

**Authors:** J. Mierzejewska, A. A. Bartosik, M. Macioszek, D. Płochocka, C. M. Thomas, G. Jagura-Burdzy

**Affiliations:** 1 The Institute of Biochemistry and Biophysics, PAS, Pawinskiego 5A, 02-106 Warsaw, Poland; 2 School of Biosciences, The University of Birmingham, Edgbaston, Birmingham B15 2TT, UK

## Abstract

The ParB protein of *Pseudomonas aeruginosa* is important for growth, cell division, nucleoid segregation and different types of motility. To further understand its function we have demonstrated a vital role of the hydrophobic residues in the C terminus of ParB*_P.a._*. By *in silico* modelling of the C-terminal domain (amino acids 242–290) the hydrophobic residues L282, V285 and I289 (but not L286) are engaged in leucine-zipper-like structure formation, whereas the charged residues R290 and Q266 are implicated in forming a salt bridge involved in protein stabilization. Five *parB* mutant alleles were constructed and their functionality was defined *in vivo* and *in vitro*. In agreement with model predictions, the substitution L286A had no effect on mutant protein activities. Two ParBs with single substitutions L282A or V285A and deletions of two or seven C-terminal amino acids were impaired in both dimerization and DNA binding and were not able to silence genes adjacent to *parS*, suggesting that dimerization through the C terminus is a prerequisite for spreading on DNA. The defect in dimerization also correlated with loss of ability to interact with partner protein ParA. Reverse genetics demonstrated that a *parB* mutant producing ParB lacking the two C-terminal amino acids as well as mutants producing ParB with single substitution L282A or V285A had defects similar to those of a *parB* null mutant. Thus so far all the properties of ParB seem to depend on dimerization.

## Introduction

The essential role of *par* loci in plasmid partitioning has long been appreciated (Williams & Thomas, 1992), while the function of chromosomally encoded *par* loci in the segregation of bacterial chromosomes is less clear. The chromosomally encoded *par* loci are highly conserved and belong to type I partitioning systems (Gerdes *et al.*, 2000). Besides the high level of identity in amino acid sequences of the chromosomal homologues of ParA and ParB, the *parS* sequences are also extremely well conserved at least between so called primary chromosomes. The localization of the *parAB* genes, as well as the majority of *parS* sites, in close vicinity to the *oriC* region could indicate a role of *par* systems in replication/segregation of chromosomes, as genes known to be crucial for these processes are situated within the *oriC* domain (20 % of the chromosome around *oriC*). Moreover, the chromosomal *par* systems are able to promote active segregation and stabilization of otherwise unstable replicons even in heterologous host cells (Bartosik *et al.*, 2004; Godfrin-Estevenon *et al.*, 2002; Lin & Grossman, 1998; Yamaichi & Niki, 2000). Although these features of chromosomally encoded *par* loci suggest that they should play a similar biological function in chromosome segregation to that which plasmid *par* systems play for plasmid DNA, studies on *par* mutants in different bacteria have revealed a more complex picture. With the exception of *Caulobacter crescentus*, the chromosomal *parA* and *parB* genes are not essential for cell viability (Mohl & Gober, 1997). However, mutations in the *parA* (*soj*) and *parB* (*spo0J*) genes lead to defects in the sporulation of *Bacillus subtilis* (Cervin *et al.*, 1998; Quisel *et al.*, 1999; Quisel & Grossman, 2000) and *Streptomyces coelicolor* (Jakimowicz *et al.*, 2002, 2006, 2007; Kim *et al.*, 2000) and in vegetative chromosome partitioning of *B. subtilis* (Ireton *et al.*, 1994), *Pseudomonas aeruginosa* (Lasocki *et al.*, 2007; Bartosik *et al.*, 2009) and *Pseudomonas putida* (Lewis *et al.*, 2002; Godfrin-Estevenon *et al.*, 2002). Several studies have indicated a role for *par* genes in origin localization and segregation (Bowman *et al.*, 2008; Ebersbach *et al.*, 2008; Figge *et al.*, 2003; Fogel & Waldor, 2006; Glaser *et al.*, 1997; Jakimowicz *et al.*, 2007; Lee *et al.*, 2003; Ptacin *et al.*, 2010; Saint-Dic *et al.*, 2006; Sharpe & Errington, 1996; Toro *et al.*, 2008; Viollier *et al.*, 2004), in the separation of sister origins and in the regulation of replication (Kadoya *et al.*, 2011; Lee & Grossman, 2006; Murray & Errington, 2008; Ogura *et al.*, 2003; Scholefield *et al.*, 2011; Webb *et al.*, 1997), structural maintenance of chromosomes (SMC), complex recruitment to the nucleoid (Gruber & Errington, 2009; Sullivan *et al.*, 2009) or in cell division initiation and cell cycle coordination (Autret & Errington, 2003; Figge *et al.*, 2003; Jakimowicz *et al.*, 2007; Marston & Errington, 1999; Mohl *et al.*, 2001; Real *et al.*, 2005; Schofield *et al.*, 2010; Thanbichler & Shapiro, 2006).

In *Pseudomonas aeruginosa*, a facultative pathogen, the *par* locus has been identified ~8 kb from *oriC*; eight out of ten *parS* sequences are located in the *ori* domain. The ParABS system of *P. aeruginosa* can stabilize an unstable plasmid in *Escherichia coli* (Bartosik *et al.*, 2004). Neither ParA nor ParB of *P. aeruginosa* is essential for the viability of the cells. Their lack causes visible phenotypic defects (more severe in the *parA* mutant) which include an over 200-fold increase in the number of anucleate cells, longer cells, slower growth rate and perturbations in colony formation and motilities (Bartosik *et al.*, 2009; Lasocki *et al.*, 2007). The increased frequency of chromosome loss observed in actively dividing cells of a *P. aeruginosa parB* mutant (Bartosik *et al.*, 2009) has been also reported for *P. putida parB* mutants (Godfrin-Estevenon *et al.*, 2002; Lewis *et al.*, 2002), but only in the transient and stationary phase of culture growth. The other phenotypic defects (motility defects, changed colony morphology, increase in cell size) caused by the lack of ParB*_P.a._* seem to be unique among ParB representatives and so far unexplained.

In terms of molecular functions, previous studies (Bartosik *et al.*, 2004) have shown that ParB of *P. aeruginosa* conforms to the behaviour of other chromosome- and plasmid-encoded homologues. It demonstrates the ability to interact with ParA and to dimerize and to bind centromere-like sequences (*parS*). ParB*_P.a._* has the ability to spread on DNA and silence genes adjacent to the *parS* sites and it has been shown that this effect is dependent on a putative helix–turn–helix (H–T–H) motif, the ability of ParB to dimerize and also on an intact N terminus (Bartosik *et al.*, 2004; Kusiak *et al.*, 2011). ParB also forms regularly distributed foci in *P. aeruginosa* cells which co-localize with the nucleoid and undergo dynamic changes (Bartosik *et al.*, 2009). In this study, we dissected the C terminus of ParB from *P. aeruginosa* demonstrating the vital role of the hydrophobic residues and C-tip of the protein in dimerization and all known functions of this protein.

## Methods

### 

#### Bacterial strains and growth conditions.

*Escherichia coli* strains used were DH5α [F*^−^*(ϕ*80*d*lacZ*ΔM15) *recA1 endA1 gyrA96 thi-1 hsdR17*(${\text{r}}_{\text{K}}^{-}{\text{ m}}_{\text{K}}^{-}$) *supE44 relA1 deoR* Δ(*lacZYA-argF*)*U196*], BL21 [F^−^ *ompT hsdS*_B_ (${\text{r}}_{\text{B}}^{-}{\text{ m}}_{\text{B}}^{-}$) *gal dcm* (λ DE3)] (Novagen), BTH101 [F*^−^*, *cya-99 araD139 galE15 galK16 rpsL1* (Sm^R^) *hsdR2 mcrA1 mcrB1*] (Karimova *et al.*, 1998) and S17-1 (*pro hsdR hsdM recA* Tp^R^ Sm^R^ ΩRP4-Tc : : Mu-Km : : Tn*7*) (Simon *et al.*, 1986). *P. aeruginosa* strains used were PAO1161 (*leu^−^*, *r^−^*, *m^+^*), kindly provided by B. M. Holloway (Monash University, Clayton, Victoria, Australia) and its derivatives: PAO1161 Rif^R^ (Lasocki *et al.*, 2007), PAO1161 Rif^R^ *parB1–18* : : Tc^R^ (*parB* null) (Bartosik *et al.*, 2009), PAO1161 Rif^R^ *parB1–288*, PAO1161 Rif^R^ *parB1–283*, PAO1161 Rif^R^ *parB282* and PAO1161 Rif^R^ *parB285* (all this work).

Bacteria were grown in Luria broth (Kahn *et al.*, 1979) at 37 or 30 °C or on Luria agar (Luria broth with 1.5 % w/v agar) supplemented with antibiotics as appropriate: benzylpenicillin sodium salt at 150 µg ml^−1^ in liquid medium and 300 µg ml^−1^ on agar plates for penicillin resistance in *E. coli*, kanamycin sulphate at 50 µg ml^−1^ for kanamycin resistance in *E. coli*, carbenicillin at 300 µg ml^−1^ for carbenicillin resistance in *P. aeruginosa*, rifampicin at 300 µg ml^−1^ for rifampicin resistance in *P. aeruginosa*. Some experiments were performed in M9 minimal medium (Sambrook *et al.*, 1989) and on MacConkey Agar Base (Difco) supplemented with 1 % maltose. The Luria agar used for blue/white screening contained 0.1 mM IPTG and 40 µg X-Gal ml^−1^.

#### Plasmid DNA isolation, analysis, cloning and manipulation of DNA.

Plasmid DNA was isolated and analysed by standard procedures (Sambrook *et al.*, 1989). The plasmids used in this study are listed in Table 1. Standard PCR (Mullis *et al.*, 1986) was performed as described previously (Lasocki *et al.*, 2007) with the primers listed in Table S1 (available with the online version of this paper). PCR site-directed mutagenesis (modified method of Stratagene, QuikChange Site-Directed Mutagenesis) was used to make *parB* alleles producing ParBs with single amino acid substitutions: L282A, V285A and L286A. In this procedure, supercoiled double stranded plasmid DNA with *parB_P.a._* (either pKLB2 or pKLB2.8) and pairs of synthetic oligonucleotide primers #21 and #22, #23 and #24 and #25 and #26 containing the desired mutation (Table S1) were used. The mutagenic oligonucleotide primers were designed to either introduce or remove the restriction site. The plasmid DNA was sequenced to verify the presence of the mutation.

**Table 1.** List of plasmids used in this work

Plasmid	Relevant features	Reference or source
pABB811	pGB2 with *parS_2/3_* sequence	Bartosik *et al.*, (2004)
pAKE600	*ori*_MB1_, *oriT*_RK2_, Ap^R^, *sacB*	El-Sayed *et al.*, (2001)
pET28mod	*ori*_MB1_, Km^R^, T*7p*, *lacO*, His tag, no *Bam*HI site, T7 tag deleted	G. Jagura-Burdzy, Warsaw, Poland
pBBR1MCS	IncA/C broad-host-range cloning vector, *lacZα*-MCS, *mob*, T*7p*, T*3p*, Cm^R^	Kovach *et al.* (1995)
pGB2	*ori*_SC101_, Sp^R^/Sm^R^, *repA* gene downstream of MCS	Churchward *et al.* (1984)
pGBT30	*ori*_MB1_, Ap^R^, *lacI*^q^, *tacp* expression vector	Jagura-Burdzy *et al.* (1991)
pGEM-T Easy	*ori*_MB1_, Ap^R^	Promega
pJMB500	pBBR1MCS with *lacI^q^ tacp-parB*	Lasocki *et al.* (2007)
pKLB2	pGBT30 with *tacp-parB*	Bartosik *et al.* (2004)
pKLB2.8	pET28mod with T*7p*-*parB*	Bartosik *et al.* (2004)
pKT25	*ori*_p15_, Km^R^, *lacp*-*cya*T25	Karimova *et al.* (1998)
pKT25-zip	Derivative of pKT25 in which the leucine zipper of GCN4 is translationally fused with *cya*T25 fragment	Karimova *et al.* (1998)
pLKB2	pKT25 modified with MCS	L. Kusiak, Warsaw, Poland
pLKB220	pLKB2 with translationally fused *cya*T25-*parA*	L. Kusiak, Warsaw, Poland
pLKB233	pLKB2 with translationally fused *cya*T25*-parB*	L. Kusiak, Warsaw, Poland
pLKB4	pUT18C modified with MCS	L. Kusiak, Warsaw, Poland
pLKB433	pLKB4 with translationally fused *cya*T18-*parB*	L. Kusiak, Warsaw, Poland
pUT18C	*ori*_ColE1_, Ap^R^, *lacp*-*cya*T18	Karimova *et al.* (1998)
pUT18C-zip	Derivative of pUT18C in which the leucine zipper of GCN4 is translationally fused with *cya*T18 fragment	Karimova *et al.* (1998)
pJMB26	*parB1–288* allele PCR amplified using #1 and #4 primers	This study
pJMB27	*parB1–283* allele PCR amplified using #1 and #5 primers	This study
pJMB28	379 bp fragment PCR amplified using #11 and #12 primers	This study
**pET28mod derivatives**		
pJMB100	pKLB2.8 derivative T7-*parB282* (site-directed mutagenesis with pair of primers #21 and #22 to introduce substitution L282A into ParB)	This study
pJMB101.1	pKLB2.8 derivative T7-*parB285* (site-directed mutagenesis with pair of primers #23 and #24 to introduce substitution V285A into ParB)	This study
pJMB102	pKLB2.8 derivative T7-*parB286* (site-directed mutagenesis with pair of primers #25 and #26 to introduce substitution L286A into ParB)	This study
pMMB5.2	pKLB2.8 derivative T7-*parB1–288* (inserted *Eco*RI–*Sal*I fragment of pJMB26)	This study
pMMB6.2	pKLB2.8 derivative T7-*parB1–283* (inserted *Eco*RI–*Sal*I fragment of pJMB27)	This study
**pAKE600 derivatives (suicide vector for gene exchange)**		
pJMB400	*Eco*RI–*Sal*I fragment of pMMB5.2 carrying *parB1–288*	This study
pJMB401	*Eco*RI–*Sal*I fragment of pMMB6.2 carrying *parB1–283*	This study
pJMB402	379 bp *Sal*I–*Bam*HI fragment of pJMB28 inserted into pJMB400	This study
pJMB403	379 bp *Sal*I–*Bam*HI fragment of pJMB28 inserted into pJMB401	This study
pJMB404	*Eco*RI–*Sal*I fragment of pJMB100 carrying *parB282*	This study
pJMB405	*Eco*RI–*Sal*I fragment of pJMB101.1 carrying *parB285*	This study
pJMB406	379 bp *Sal*I–*Bam*HI fragment of pJMB28 inserted into pJMB404	This study
pJMB407	379 bp *Sal*I–*Bam*HI fragment of pJMB28 inserted into pJMB405	This study
**pBBR1MCS1 derivatives**		
pJMB501	*Bam*HI–*Sal*I fragment of pJMB604 carrying *lacI^q^* and *tacp–parB1–283* transcriptional fusion	This study
pJMB502	*Bam*HI–*Sal*I fragment of pJMB603 carrying *lacI^q^* and *tacp–parB1–288* transcriptional fusion	This study
pJMB503	*Bam*HI–*Sal*I fragment of pJMB600 carrying *lacI^q^* and *tacp–parB282* transcriptional fusion	This study
pJMB504	*Bam*HI–*Sal*I fragment of pJMB601.1 carrying *lacI^q^* and *tacp–parB285* transcriptional fusion	This study
**pGBT30 derivatives**		
pJMB600	*Eco*RI–*Sal*I fragment of pJMB100 to form a *tac–parB282* transcriptional fusion	This study
pJMB601.1	pKLB2 derivative *tacp–parB285* (PCR site-directed mutagenesis with pair of primers #23 and #24 to introduce V285A substitution into ParB)	This study
pJMB602	*Eco*RI–*Sal*I fragment of pJMB102 to form a *tacp–parB286* transcriptional fusion	This study
pJMB603	*Eco*RI–*Sal*I fragment of pJMB26 to form a *tacp–parB1–288* transcriptional fusion	This study
pJMB604	*Eco*RI–*Sal*I fragment of pJMB27 to form a *tacp–parB1–283* transcriptional fusion	This study
**BACTH system plasmids (pUT18C derivatives)**		
pJMB700	*Eco*RI–*Hin*cII fragment of pJMB100 inserted into pLKB4 between restriction sites *Eco*RI and *Sma*I to create a *cyaT18*–*parB282* translational fusion	This study
pJMB701.1	*Eco*RI–*Hin*cII fragment of pJMB101.1 inserted into pLKB4 between restriction sites *Eco*RI and *Sma*I to create a *cyaT18*–*parB285* translational fusion	This study
pJMB702	*Eco*RI–*Hin*cII fragment of pJMB102 inserted into pLKB4 between restriction sites *Eco*RI and *Sma*I to create a *cyaT18*–*parB286* translational fusion	This study
pJMB703	*Eco*RI–*Hin*cII fragment of pMMB5.2 inserted into pLKB4 between restriction sites *Eco*RI and *Sma*I to create a *cyaT18*–*parB1–288* translational fusion	This study
pJMB704	*Eco*RI–*Hin*cII fragment of pMMB6.2 inserted into pLKB4 between restriction sites *Eco*RI and *Sma*I to create a *cyaT18*–*parB1–283* translational fusion	This study

#### Bacterial transformation.

Competent cells of *E. coli* were prepared by using a standard CaCl_2_ method (Sambrook *et al.*, 1989). Transformation of *P. aeruginosa* strains was done according to a previously published method (Irani & Rowe, 1997).

#### Introduction of *par* mutant alleles into *P. aeruginosa* PAO1161.

Mutant *parB* alleles were cut out as *Eco*RI–*Sal*I fragments from pMMB5.2, pMMB6.2, pJMB100 and pJMB101.1 and inserted into pAKE600, suicide vector unable to replicate in *P. aeruginosa* strains (El-Sayed *et al.*, 2001), to create pJMB400, pJMB401, pJMB404 and pJMB405, respectively. To provide the region of homology downstream of truncated *parB*s the 389 bp DNA fragment corresponding to genomic DNA adjacent to the 3′ end of *parB* was amplified by PCR with the pair of primers #11 and #12 (Table S1) and then introduced as a *Sal*I–*Bam*HI fragment into pJMB400, pJMB401, pJMB404 and pJMB405 to form pJMB402, pJMB403, pJMB406 and pJMB407, respectively.

Transformants of *E. coli* strain S17-1 with pAKE600 derivatives were used as the donors in conjugation with the recipient strain *P. aeruginosa* PAO1161 Rif^R^. The transconjugants with pAKE600 derivatives integrated into the chromosome were treated as described previously (Lasocki *et al.*, 2007). The PCR products (chromosomal DNA isolated from the putative mutants as the templates with primers #1 and #12) were screened by *Sal*I digestion and finally the presence of modifications was confirmed by sequencing of PCR fragments.

#### ‘Silencing’ assay.

*E. coli* DH5α(pABB811*parS_2/3_*) and DH5α(pGB2) cells were transformed with the appropriate pGBT30*tacp-parB* derivatives. Undiluted and 10- and 100-fold dilutions of the initial transformation mixture were plated to select for incoming plasmid (Luria agar supplemented with penicillin) or for both incoming and resident plasmid (Luria agar with penicillin and streptomycin) with and without 0.5 mM IPTG to induce ParB production. After 24 h incubation at 37 °C the colonies were counted and the number of different class transformants in the original transformation mixture was estimated.

#### Purification of His_6_-tagged proteins.

*E. coli* strain BL21(DE3) was transformed with pET28mod derivatives encoding histidine-tagged (MGSS*HHHHHH*SSGLVPRGSHSEF) ParB derivatives and protein overexpression and purification was carried out as described previously (Bartosik *et al.*, 2004).

#### Cross-linking with glutaraldehyde.

His_6_-tagged polypeptides purified on Ni^2+^-agarose columns (at 0.1 mg ml^−1^) were cross-linked by use of glutaraldehyde (Jagura-Burdzy & Thomas, 1995) and separated on 10 % (w/v) SDS-PAGE gels. The proteins were transferred onto nitrocellulose membranes and Western blot analysis was performed with anti-ParB antibodies, as described previously (Bartosik *et al.*, 2004).

#### Analysis of protein–DNA interactions by electrophoretic mobility shift assay (EMSA).

To check the ability of mutated ParB*_P.a._* proteins to bind *parS* DNA *in vitro*, a nonradioactive EMSA (Leonard *et al.*, 2004) was performed. A 16 bp dsDNA fragment (annealed oligonucleotides, #17 and #18, Table S1) containing the *parS_2/3_* (Bartosik *et al.*, 2004) was used in EMSA. Samples (5.6 pmol) of *parS_2/3_* oligonucleotides without or with increased concentration of purified His_6_-tagged ParB and its derivatives were incubated under conditions described previously (Kusiak *et al.*, 2011). Negative control of the binding reaction was provided by use of unrelated dsDNA with palindromic sequence (annealed primers #19 and #20, Table S1) and the same amounts of ParB in the incubation mixture. The samples were analysed on 10 % (w/v) non-denaturing polyacrylamide gel in TBE buffer (Sambrook *et al.*, 1989). DNA bands were stained with 0.5 µg ethidium bromide ml^−1^ and visualized on a UV transilluminator.

#### Growth experiments and sample preparation for Western blotting.

The growth of bacteria was monitored by measuring OD_600_; the cultures were diluted and plated on Luria agar to establish c.f.u. ml^−1^. Bacteria were harvested, resuspended in sonication buffer (50 mM phosphate buffer, pH 8.0, and 300 mM NaCl) and disrupted by sonication. Crude extracts from the same number of cells were analysed by SDS-PAGE followed by Western blotting performed as described previously (Bartosik *et al.*, 2004).

#### Bacterial adenylate cyclase two-hybrid system (BACTH system).

The interactions between ParB mutant derivatives and either wild-type ParB or ParA were analysed by using the bacterial two-hybrid system BACTH (Karimova *et al.*, 1998). The C-terminal *parB* mutant alleles have been cloned as *Eco*RI–*Hin*cII fragments from pET28mod derivatives into pLKB4 (derivative of pUT18C) to create translational fusions with the T18 catalytic domain of *Bordetella pertussis* adenylate cyclase, CyaT18-ParB. The wild-type *parA* and wild-type *parB* alleles have been cloned into pLKB2 (modified pKT25) to produce translational fusions: CyaT25-ParA and CyaAT25-ParB. *E. coli* BTH101 *cya* strain was co-transformed with both pLKB4 and pLKB2 derivatives and plated on indicator MacConkey base medium supplemented with 1 % maltose (as the only carbon source), penicillin, kanamycin and 0.5 mM IPTG. The plates were incubated for 48 h at 27 °C.

#### Motility assays.

The swimming, swarming and twitching assays were performed according to the method of Rashid & Kornberg (2000) with modifications described previously (Lasocki *et al.*, 2007). All sets of plates were standardized by using the same volume of medium.

#### DAPI staining and immunofluorescence microscopy.

The DAPI staining procedure and immunofluorescence microscopy were carried out as previously described (Bartosik *et al.*, 2004; Bignell *et al.*, 1999). Cells were examined using an Eclipse E800 light fluorescence microscope (Nikon) fitted with an ORCA ER CCD camera (Hamamatsu). Images were captured and manipulated on PC Windows XP Professional PL with the Lucia General 5.0 (Laboratory Imaging).

#### *In silico* ParB*_P.a._* dimer modelling.

Amino acids sequences of ParB of *P. aeruginosa*, ParB of *P. putida*, KorB of RK2/RP4 (IncP-1α) and R751 (IncP-1β) were aligned using MAFT (Katoh & Toh, 2008), clustal w (Larkin *et al.*, 2007) and T-Coffee (Notredame *et al.*, 2000) servers, and manually adjusted. A structural model of the monomeric C terminus of *P. aeruginosa* ParB was obtained using Sybyl-x1.1 package (TRIPOS) on the basis of ParB*_P.a._* and KorB_RP4_ alignment (Fig. S1) and KorB_RP4_ crystal structure (Delbrück *et al.*, 2002). The structure of the C-terminal dimer of ParB*_P.a._* (superposition on KorB dimer) was subjected to energy minimization (100 steps) using the AmberFF99 force field as implemented in Sybyl-x1.1.

## Results

### Predicting amino acids essential for ParB dimerization

A comparison of ParB*_P.a._* (290 amino acids) with other chromosomal homologues revealed highly conserved segments designated BoxI (S66–R79) and BoxII (Y86–A97) (Yamaichi & Niki, 2000), a H–T–H motif and regions 1 to 4 (R6–L16, L123–A138, V211–L224, G270–I289, respectively) (Bartosik *et al.*, 2004) (Fig. S2). Previous studies on ParB*_P.a._* using *in vivo* and *in vitro* methods (Bartosik *et al.*, 2004) identified a C-terminal fragment of 56 amino acids (ParB235–290) as the dimerization domain for ParB*_P.a._* and indicated that deletion of the last seven residues from this domain (yielding ParB235–283) abolished its dimerization.

**Fig. 1.**
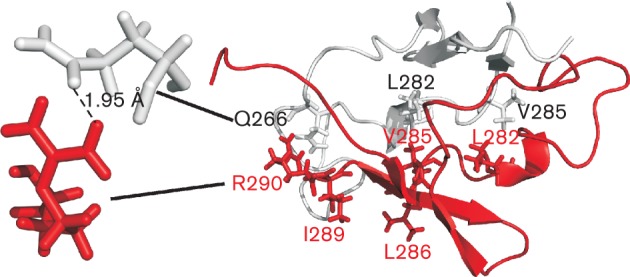
Model of a dimer of C termini of ParB*_P.a._* (amino acids 242–290). The mutagenized residues are shown as sticks in the red subunit (labelled according to their position in the ParB*_P.a._* sequence). The indicated residues L282 and V285 in the grey subunit are possibly involved in a leucine zipper formation. The distance between R290 of one monomer and Q266 of another facilitates the electrostatic interactions (magnification at the left).

The 3D structure of ParB*_P.a._* has not yet been solved. The sequence of the C terminus of ParB*_P.a._* aligns well with the C-terminal part of the ParB homologue – KorB of RK2 (IncP-1α) (Fig. S1) and moreover the two domains are functionally interchangeable. Replacement of 61 amino acids from the C terminus of ParB by the C-terminal 100 amino acids of KorB (ParB1–229-KorB258–358) restores its ability to dimerize and bind *parS* with high affinity *in vitro* as well as to transcriptionally silence genes near a *parS* site *in vivo* (Bartosik *et al.*, 2004). Therefore, on the basis of crystallographic analysis of the C-terminal part of KorB of RK2/RP4 (Delbrück *et al.*, 2002) a model of the C-terminal domain of ParB*_P.a._* was built *in silico* (Fig. 1). According to this model the hydrophobic residues L282, V285 and I289 (but not L286) are engaged in a leucine-zipper-like structure, whereas the charged R290 and Q266 are implicated in forming a salt bridge involved in stabilization of the ParB dimer. To verify this model, two alleles of *parB* with C-terminal deletions of either seven (*parB1–283*) or two amino acids, I289 and R290, (*parB1–288*) were amplified by PCR and three alleles coding for ParBs each with a single amino acid substitution – L282A, V285A and L286A – were constructed by applying PCR site-directed mutagenesis. These alleles were introduced into appropriate vectors and their products were tested for the ability to dimerize, bind DNA *in vitro*, spread on DNA and interact with ParA *in vivo*.

### Spreading on DNA *in vivo* – ‘silencing test’ in *E. coli*

The ParB*_P.a._* protein recognizes the *parS* sequence as a dimer then self-associates and spreads on DNA causing transcriptional silencing of genes adjacent to *parS* (Bartosik *et al.*, 2004). The plasmid pGB2 (Churchward *et al.*, 1984) used for the ‘silencing test’ is an Sm^R^ stable replicon based on pSC101 in which a multiple cloning site (MCS) is inserted approximately 200 bp upstream of the promoter for the initiator gene *repA*. The presence of *parS* close to the *repA* promoter in pABB811 does not influence plasmid stability unless wild-type ParB is produced *in trans* from pKLB2 (*tacp-parB*) (Bartosik *et al.*, 2004). The transformation frequency of *E. coli* DH5α(pABB811) with pKLB2 in the absence of IPTG with selection for incoming and resident plasmid was two- to threefold lower than the transformation frequency when only the incoming plasmid is selected. Addition of 0.5 mM IPTG to transformation plates with double selection (conditions of ParB overproduction) decreases the number of transformants more than 100-fold (Table 2) in comparison with the number of transformants grown on double selection plates without IPTG.

**Table 2.** Transformation frequencies of DH5α(pABB811*parS*) strain with plasmids overexpressing various *parB_P.a._* alleles The experiments were repeated three times; the same pattern of ‘silencing’ was observed.

Plasmid used for transformation	Selection plate		
	L agar+Pn	L agar+Pn Sm	L agar+Pn Sm IPTG
pGBT30 (vector)	4.47×10^4^	3.90×10^4^	4.07×10^4^
pKLB2 (wt *parB*)	2.40×10^3^	9.00×10^2^	<10
pJMB600 (*parB282*)	8.60×10^3^	5.80×10^3^	3.50×10^3^
pJMB601.1 (*parB285*)	2.18×10^4^	9.70×10^3^	6.80×10^3^
pJMB602 (*parB286*)	1.30×10^3^	5.00×10^2^	<10
pJMB603 (*parB1–288*)	1.20×10^4^	4.80×10^3^	4.10×10^3^
pJMB604 (*parB1–283*)	4.30×10^3^	1.80×10^3^	1.50×10^3^

The silencing test was repeated to establish the effect of overproducing the modified ParB proteins on the stability of pABB811 in *E. coli* DH5α(pABB811). The numbers of transformants with selection for either incoming plasmid (Pn) or both incoming and the resident plasmids (Pn Sm) with and without IPTG present are shown in Table 2. Only ParBL286A (pJMB602) caused significant instability of pABB811 and a loss of streptomycin resistance of the recipient strain when ParB was overproduced during growth with IPTG (more than 100-fold decrease in the number of transformants on Pn Sm IPTG plates, effect observed for wild-type ParB delivered from pKLB2). The other plasmids tested had very little impact on stability of pABB811 (two- to threefold decrease in the number of double transformants grown in the presence of inducer in comparison with the number of transformants selected for incoming plasmid). The deletions of seven (pJMB604) or two amino acids I289 and R290 from the C terminus (pJMB603) as well as the single amino acid substitutions V285A or L282A impaired the silencing property of ParB. Western blotting on extracts from analysed transformants has shown a level of ParB overproduction for all mutant derivatives similar to wild-type ParB (Fig. S3).

### ParB*_P.a._* dimerization *in vitro*

**Fig. 2.**
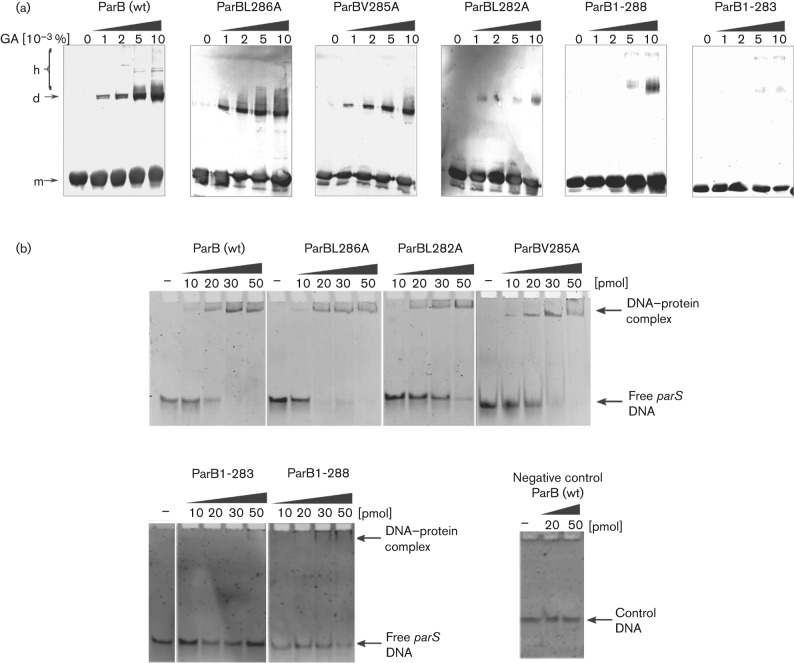
ParB*_P.a._* self-association and DNA binding *in vitro*. (a) Cross-linking with glutaraldehyde (GA) of ParB variants. Purified His_6_-tagged proteins (0.1 mg ml^−1^) were incubated at room temperature for 20 min without (0) or with increasing concentrations (1×, 2×, 5× and 10×10^−3^ %) of glutaraldehyde. The samples were separated by SDS-PAGE on 12 % gels and analysed by Western blotting with anti-ParB antibodies. Monomeric, dimeric and higher forms are indicated by m, d and h, respectively. (b) DNA binding affinity of ParB derivatives (EMSA). Purified His_6_-tagged proteins (10, 20, 30 and 50 pmol) were incubated with 5.6 pmol double-stranded *parS_P.a._* oligonucleotide at 37 °C for 15 min. As a control, double-stranded oligonucleotides with an unrelated palindromic motif were used under the same conditions.

For the *in vitro* analysis, wild-type ParB as well as modified ParB proteins (ParB1–283, ParB1–288, ParBL282A, ParBV285A and ParBL286A) with a His_6_ tag attached to the N terminus were expressed upon induction with IPTG from pET28mod derivatives in *E. coli* strain BL21(DE3). All ParB variants were present in the soluble cellular fraction and it was possible to purify them in a native form on a Ni^2+^-agarose column. Wild-type ParB*_P.a._* protein has previously been found to dimerize and form higher order complexes *in vitro* (Bartosik *et al.*, 2004). The dimerization and oligomerization domains are separate in ParB*_P.a._* (Kusiak *et al.*, 2011). The purified His_6_-tagged ParB derivatives were treated with increasing concentrations of the cross-linking agent glutaraldehyde (GA). Wild-type ParB protein dimerized so strongly that even at the lowest glutaraldehyde concentration (0.001 %) dimeric species were visible. The monomeric and dimeric forms were predominant after SDS-PAGE separation and Coomassie blue staining (not shown). To visualize higher-order complexes, a Western blot analysis was performed with anti-ParB antibodies (Fig. 2a). ParBL286A dimerized and formed higher order complexes as efficiently as the wild-type protein. ParBL282A and ParBV285A dimerized but with much lower effectiveness than wild-type ParB. The C-truncated ParBs – ParB1–283 and ParB1–288 – were drastically impaired in dimerization. These results confirmed that the C-terminal fragment of ParB is the major determinant of its ability to form dimers, as deletion of seven (ParB1–283) or even two (ParB1–288) amino acids impaired significantly the ability of monomers to interact.

### ParB*_P.a._* DNA binding *in vitro* (EMSA)

A previous study (Bartosik *et al.*, 2004) indicated that self association of ParB is important for efficient DNA binding (deletion of the C terminus in ParB1–229 significantly decreased the DNA binding ability but did not completely stop it from binding *parS*). All purified ParB*_P.a._* derivatives were tested for binding to *parS_P.a._* using a standard EMSA (Fig. 2b). Non-radioactive EMSA was performed on *parS* (annealed oligonucleotides #17 and #18) and an unrelated palindrome motif (annealed oligonucleotides #19 and #20) as a control. Wild-type ParB did not bind the control oligonucleotides at tested concentrations. ParBL286A showed affinity towards *parS* approximately twofold higher than wild-type ParB whereas ParBV285A and ParBL282A bound *parS* but with twofold lower affinity. ParB1–288 and ParB1–283 hardly shifted the double-stranded *parS* oligonucleotides at tested concentrations. Therefore the ability to bind *parS* seems to correlate with the degree of dimerization proficiency as illustrated by comparing Fig. 2(a) and (b).

### ParB*_P.a._* self association and interaction with ParA*_P.a._ in vivo*

To check the interactions of mutated ParB proteins with wild-type ParB and ParA *in vivo*, the bacterial adenylate cyclase two-hybrid system in *E. coli* (Karimova *et al.*, 1998, 2000) was applied. Mutated ParB derivatives were translationally fused to CyaT18 fragment (pUT18C derivatives), whereas wild-type ParB and ParA were fused to CyaT25 fragment (pKT25 derivatives). *E. coli* BTH101, an adenylate-cyclase-deficient strain (*cya*), was co-transformed with a mixture of appropriate pairs of BACTH system plasmids and plated on MacConkey base medium supplemented with 1 % (w/v) maltose, 0.5 mM IPTG and selective antibiotics.

**Fig. 3.**
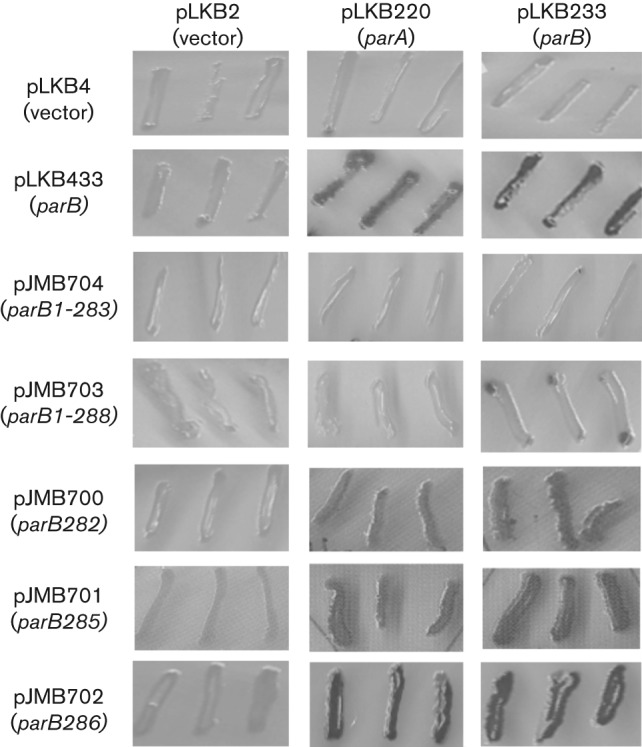
Heterodimer formation of ParB variants with wild-type ParB and ParA *in vivo* (BACTH). Double transformants of *E. coli* BTH101(pLKB4 derivatives)(pLKB2 derivatives) were streaked on MacConkey indicator medium supplemented with penicillin, kanamycin and 0.5 mM IPTG to visualize protein interactions. Dark streaks are indicative of interaction between the two proteins, whereas light streaks correspond to a lack of interaction.

The results of *in vivo* BACTH analysis confirmed the conclusions from the *in vitro* dimerization studies presented above. The two short deletion mutants ParB1–283 and ParB1–288 were unable to associate with wild-type ParB whereas interactions between ParBL286A and wild-type ParB were similar to self-association of wild-type ParB as demonstrated by BTH101 (pLKB702)(pLKB233) transformants (Fig. 3). Interactions of ParBL282A and ParBV285A with wild-type ParB were weaker than control interactions between pLKB433 and pLKB233 but still very clear. The analysis of interactions of mutant ParB derivatives and ParA showed a correlation between the efficiency of ParB dimerization and the ability to interact with ParA in the BACTH system. ParBL286A demonstrated interactions with ParA similar to those for wild-type ParB. Visibly weaker interactions between ParA and ParBL282A or ParBV285A and no interactions between ParA and ParB1–283 or ParB1–288 were detected. This strongly suggests that the dimer form of ParB is required for interactions with ParA.

### Introduction of mutant *parB* alleles into the *P. aeruginosa* chromosome

**Fig. 4.**
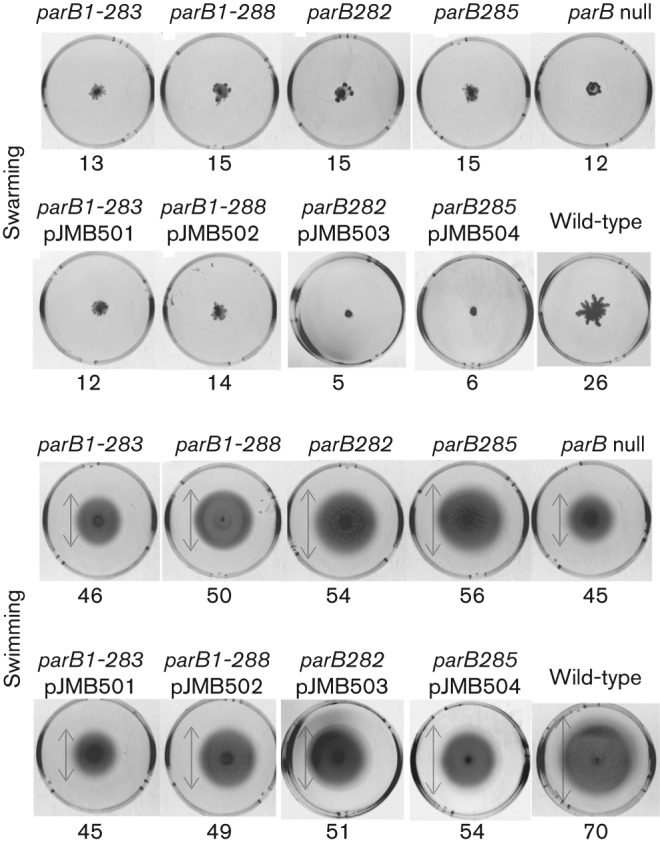
Motility assays for *P. aeruginosa* PAO1161 Rif^R^, *parB* mutants and merodiploid strains. Volume-standardized plates for swarming and swimming were inoculated with a sterile toothpick using material from a single colony and incubated for 24 h at 30 °C. The zones of growth/spreading are indicated in mm below the images; the boundaries of the swimming zones are marked by arrows.

The C-terminal modifications of ParB (ParB1–283 and ParB1–288 as well as ParBL282A and ParBV285A) cause defects in dimerization and in turn defects in DNA binding, transcriptional silencing and interaction with ParA. To determine the phenotypic effect of these mutations all four alleles were introduced into the *P. aeruginosa* chromosome using suicide vector pAKE600 and allele exchange via homologous recombination (El-Sayed *et al.*, 2001). The new *P. aeruginosa* PAO1161 Rif^R^*parB* mutants, *parB1–288* and *parB1–283*, *parB282* and *parB285* were tested for various forms of motility: swimming, swarming and twitching. A drastic defect in swarming, slight defect in swimming and no effect on twitching have been observed previously for the *P. aeruginosa* PAO1161 Rif^R^ *parB* null strain (Bartosik *et al.*, 2009). Neither of the new tested mutants was disturbed in twitching (data not shown) but all four were strongly impaired in swarming and slightly affected in swimming (Fig. 4). The *parB282* and *parB285* mutants demonstrated lesser defects in swimming when compared with *parB* null, *parB1–288* and *parB1–283* mutants.

To look at the effects of *parB* mutations on *P. aeruginosa* PAO1161 Rif^R^ growth, time-course experiments in both rich (Luria broth) and minimal (M9) medium were conducted. Each growth experiment was performed using cells freshly taken from a deep-frozen stock to reduce the possibility of accumulation of secondary mutations. The new PAO1161 Rif^R^*parB* mutants (short deletions and single amino acid substitutions) demonstrated changes in the growth rate similar to the *parB* null strain. They showed ~10 % longer mean generation time (mgt) in comparison with wild-type PAO1161Rif^R^ when grown in Luria broth or minimal medium (M9) at 37 °C, and ~20 % longer mgt when grown in Luria broth at 30 °C (Table 3).

**Table 3.** Phenotypes of PAO1161 Rif^R^*parB* mutants

*parB* allele	Division time (min)*			Anucleate cells (%)†	Mean cell length (µm)†
	L broth 37 °C	L broth 30 °C	M9 37 °C		
*parB1–283*	32±1	54±5	119±10	1.8	1.9±0.4
*parB1–283*/pJMB501	32±1	nd	nd	1.6	1.9±0.4
*parB1–288*	32±1	55±5	122±11	1.3	1.8±0.4
*parB1–288*/pJMB502	32±1	nd	nd	1.3	1.8±0.4
*parB282*	32±1	52±5	120±10	2.2	1.9±0.5
*parB282/*pJMB503	nd	nd	nd	2.6	1.9±0.5
*parB285*	32±1	58±5	125±10	2.2	1.8±0.4
*parB285/*pJMB504	nd	nd	nd	3.6	1.8±0.5
*parB* null	33±2	54±4	125±11	2.1	1.9±0.5
Wild-type	30±1	46±3	110±10	<0.01	1.6±0.4

* Data are from three independent experiments. nd, Not done.

† Estimated by DAPI staining and microscopic observations. Data are from at least 1000 cells.

The *parB* mutant strains were also examined for the frequency of anucleate cell formation. Bacterial cells were collected from cultures at late exponential growth phase (OD_600_ 0.8). The cells were fixed and DAPI-stained to visualize chromosomes. The number of cells without chromosomes and the mean cell length were estimated using fluorescence microscopy combined with appropriate software. The frequency of anucleate cell formation for the *parB* null mutant was more than 100-fold higher than for the wild-type strain under the same growth conditions on the sample of at least 1000 cells (Table 3). The short C-terminal deletion mutants PAO1161 Rif^R^*parB1–288*, *parB1–283* as well as the substitution mutants *parB282* and *parB285* produced anucleate cells at similar frequencies to those observed for the PAO1161 Rif^R^*parB* null mutant. Measurements of cell length showed that all *parB* mutants produce cells up to 10 % longer on average than those of the wild-type similarly to the PAO1161 Rif^R^*parB* null mutant (Table 3).

**Fig. 5.**
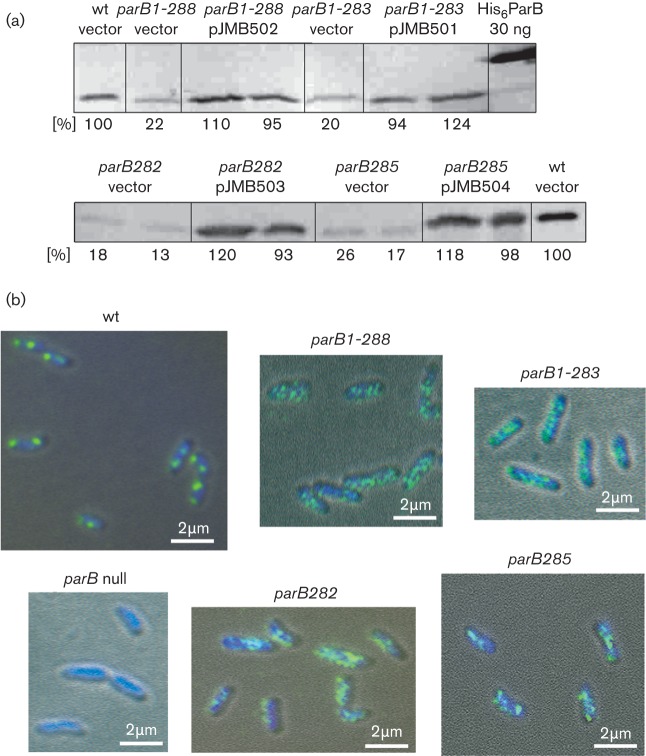
Effect of ParB modifications on its turnover and cellular localization. (a) Intracellular levels of ParB in *P. aeruginosa* PAO1161 Rif^R^ pBBR1-MCS, *parB* mutants with pBBR1-MCS and merodiploids of *parB* mutants with the appropriate mutant allele on the pBBR1-MCS under control of *tacp*. Total cellular extracts from 1×10^9^ cells were separated by SDS-PAGE and analysed by Western blotting with anti-ParB antibodies. His-tagged purified ParB was run on the gel as a control. Two cultures of all merodiploid strains were analysed. The intensities of signals were estimated using ImageQuant and shown underneath as relative (%) to the values obtained for the wild-type (wt) strain. (b) Immunofluorescence/phase-contrast overlaid images showing ParB localization in cells of *P. aeruginosa* PAO1161 Rif^R^*parB* mutants. Cells from the exponential growth phase (OD_600_ 0.5), grown on Luria broth at 37 °C, were fixed. The dark background is a phase-contrast image; blue colour shows DAPI-stained chromosome; green colour indicates FITC-stained ParB*_P.a._.*

In order to check the intracellular concentration of the mutated ParBs, equal numbers of cells of the PAO1161 Rif^R^ strain and *parB* mutants from the same growth phases were collected and analysed by Western blotting with anti-ParB antibodies. The amount of ParBs truncated at the C terminus was approximately five- to sixfold lower than the amount of wild-type ParB in actively dividing cells of the PAO1161 Rif^R^ strain. A similar decrease was observed for two ParBs with amino acid substitutions at the C terminus (ParBL282A and ParBV285A) probably due to the lower stability of monomeric ParB (Fig. 5a). To exclude the possibility that it was the decreased cellular concentration of ParB rather than the specific mutation that was responsible for the observed defects in growth and nucleoid segregation, the medium-copy-number broad-host-range plasmid pBBR1-MCS1 carrying the mutated *parB* alleles under control of *tacp* (the plasmid series from pJMB501 to pJMB504) was introduced into the appropriate chromosomal mutants. Western blotting of extracts from defined numbers of cells of such transformants grown in the absence of IPTG showed that the level of mutant ParBs was similar or even higher when related to wild-type ParB in PAO1161 grown under the same conditions (Fig. 5a). The merodiploid strains were also tested for motility (Fig. 4), growth rate and anucleate cell production (Table 3). No suppression of the defects was observed by increasing production of mutant ParB derivatives, confirming that specific changes in ParB and not a decreased level were responsible for the mutant phenotypes.

To visualize the localization and ability of the ParB mutant derivatives to form intracellular foci, immunofluorescence microscopy was applied (Fig. 5b). Fixed cells from the exponential growth phase were incubated first with purified anti-ParB antibodies and then with FITC-conjugated anti-rabbit IgG. Cells of the PAO1161 Rif^R^*parB* null mutant were also examined as a control for the specificity of the antibodies used. The majority of the actively dividing cells of the wild-type strain of *P. aeruginosa* contained from two to four regularly spaced ParB foci as expected from the number of *ori* domains. In cells of mutants *parB1–283*, *parB1–288*, *parB282* and *parB285* no such strictly organized foci were observed, but instead, multiple irregularly distributed signals appeared in the region of the nucleoid. The fluorescence signals in transformants of the PAO1161 Rif^R^*parB1–283* and PAO1161 Rif^R^*parB1–288*, in which truncated ParBs were also supplied from the plasmids, showed multiple and dispersed fluorescent foci, similar to those seen in the mutants (data not shown). The diminished ability of ParB derivatives to dimerize leads to the defect in foci compaction (probably *oriC* domain organization) and nucleoid segregation.

## Discussion

The work described in this paper adds important details to our understanding of ParB from *P. aeruginosa*, which is a key representative of the large family of ParB proteins encoded by both plasmids and chromosomes. Our earlier *in vivo* studies revealed that overproduced ParB of *P. aeruginosa* is able to silence the expression of genes adjacent to the *parS* site (Bartosik *et al.*, 2004) and that spreading activity relies on dimer formation by the C terminus, DNA binding and N-terminal polymerization domain (Bartosik *et al.*, 2004; Kusiak *et al.*, 2011). Accumulating evidence has shown that this activity is a common feature of ParB family members of type IA (Bingle *et al.*, 2005; Rodionov *et al.*, 1999; Schumacher *et al.*, 2007, 2010), also including the chromosomal homologues (Bartosik *et al.*, 2004; Breier & Grossman, 2007). This silencing is thought to be a consequence of spreading on DNA due to ParB–ParB interactions through the N-terminal polymerization domain (Kusiak *et al.*, 2011). However, the physiological role of both plasmidic (Rodionov & Yarmolinsky, 2004) and chromosomal ParBs spreading on DNA is unclear. The recent studies on Spo0J of *B. subtilis* showed that Spo0J spreads around each *parS* site on chromosomal DNA over dozens of kilobases (Breier & Grossman, 2007) but under the conditions tested, this process did not significantly affect expression of the majority of genes near *parS*, with the exception of some sporulation genes. The crystallographic studies on ParB homologues of type IA (Delbrück *et al.*, 2002; Khare *et al.*, 2004; Leonard *et al.*, 2004; Schumacher *et al.*, 2007) combined with further experimental verification should help to elucidate the exact role of ParB spreading on DNA and whether this role is universal for all ParB homologues. The discoveries of interactions of chromosomal ParB homologues with DnaA (control of initiation of replication), different proteins involved in chromosome organization and *ori* domain localization (SMC, PopZ, TipN) and cytokinesis (FtsZ, MipZ) suggest an important biological role of Par proteins in a wide spectrum of processes, some of them possibly species-specific (Bowman *et al.*, 2008; Donovan *et al.*, 2010; Ebersbach *et al.*, 2008; Gruber & Errington, 2009; Kadoya *et al.*, 2011; Murray & Errington, 2008; Ptacin *et al.*, 2010; Schofield *et al.*, 2010; Scholefield *et al.*, 2011; Sullivan *et al.*, 2009; Thanbichler & Shapiro, 2006; Toro *et al.*, 2008). In *P. aeruginosa* ParB seems to be involved not only in the chromosome segregation but also in the control of growth rate, cell motilities and colony morphology (Bartosik *et al.*, 2009). Its role in some but not all of these processes depends on interactions with its cognate ParA counterpart (M. Kusiak and G. Jagura-Burdzy, unpublished data). It was unclear whether a ParB dimer is required for interactions with ParA and other putative partners. To correlate the structural information with the physiological role of ParB we looked closely at the C-terminal domain which we had previously established to be the dimerization domain of ParB.

Although ParB*_P.a._* has not been crystallized yet, the putative 3D structure for the C-terminal (242–290 amino acids) domain of ParB*_P.a._* has been predicted, based on crystallographic data for its homologue, the KorB protein of plasmid RK2/RP4 (Delbrück *et al.*, 2002) (Fig. 1). We constructed five *parB* mutant alleles to define the functionality of proteins modified in the C-terminal region. Both *in vitro* and *in vivo* tests on the ability of ParB derivatives to dimerize indicated that the last two amino acids at the C terminus within the conserved region 4 are essential for the ability of ParB*_P.a._* to self-associate. Removal of I289 and R290 rendered ParB inactive in dimer formation. In agreement with the structural prediction *in silico* two hydrophobic residues L282 and V285 have been confirmed to play a vital role in ParB dimerization. The alanine substitution derivatives ParBL282A and ParBV285A showed detectable changes in self-association *in vitro* and in association with wild-type ParB *in vivo*. On the other hand, L286, which should be directed outwards from the putative dimer (Fig. 1), has been confirmed experimentally not to be involved in self-associations. The alanine substitution derivative ParBL286A behaved like wild-type ParB in all tests with the exception of EMSA when it seemed to bind *parS* with even higher affinity than wild-type ParB.

The four ParB mutant derivatives impaired to various extents in dimerization were also impaired to similar extents in *parS* binding, strongly implying that ParB binds to *parS* as a dimer. None of these mutants was also able to silence genes adjacent to *parS*, suggesting that dimerization through the C terminus is a prerequisite for spreading on DNA. The necessity of ParB to form dimers before interacting with its ParA partner was confirmed by analysis of mutants in the BACTH system. The observed *in vivo* heterologous interactions between ParB mutant derivatives and ParA correlated in strength with the ability of ParB mutants to interact with wild-type ParB as a dimer.

When these four *parB* alleles were introduced into the *P. aeruginosa* chromosome by allele exchange they caused defects in growth rate and motilities (swarming and swimming) and more than 100-fold increase in the frequency of anucleate cell formation. Immunofluorescence microscopy showed that in contrast with wild-type ParB, which is organized into one–four regularly distributed foci, the modified ParBs formed multiple smaller foci dispersed within the boundaries of the nucleoid.

It has been observed that all modified ParBs are present in lower quantities per cell and are more prone to degradation than wild-type ParB, probably due to their inability to be protected by ParA (Lasocki *et al.*, 2007; Bartosik *et al.*, 2009). The elevation of mutant ParB production to the level observed for wild-type ParB did not suppress the *parB* mutant phenotypes in the constructed merodiploid strains, suggesting that the decreased level of protein is not the main factor responsible for the visible deficiencies of the mutants.

Despite the fact that ParBs with single amino acid substitution (ParB282 and ParB285) seem to be significantly less impaired in dimerization, DNA binding or interactions with ParA than the truncated derivatives ParB1–283 and ParB1–288, the phenotypes of four new *parB* mutants were almost identical (with slight difference between the deletion and point mutants in swimming defects) and they resembled the phenotype of the *parB* null mutant (Bartosik *et al.*, 2009). The data presented suggest that even small changes in the dimerization ability of ParB may translate into lower affinity of *parS* binding and in turn result in inability to spread on DNA (silencing test). The spreading on DNA has been shown to determine the biological function of ParB in *P. aeruginosa* (Kusiak *et al.*, 2011).

In conclusion, an *in silico* model of the ParB*_P.a._* C-terminal dimerization domain has identified the hydrophobic residues L282 and V285 and charged residue R290 as vital for dimerization. Substitution of hydrophobic residues by alanine or removal of the two last amino acids I289 and R290 impairs ParB*_P.a._* in dimerization, *parS* binding and ParA interaction and renders it inactive in spreading on DNA (transcriptional silencing). Since such truncation of ParB as well as alanine substitution of two hydrophobic residues led to the same deficiencies in growth, genome segregation and motilities as a complete lack of ParB in *P. aeruginosa*, it is clear that dimerization is a vital prerequisite for the function of ParB in the cells.

## References

[r1] Autret S. Errington J. **(**2003**).** A role for division-site-selection protein MinD in regulation of internucleoid jumping of Soj (ParA) protein in *Bacillus subtilis*. Mol Microbiol 47, 159–169. 10.1046/j.1365-2958.2003.03264.x 12492861

[r2] Bartosik A. A. Lasocki K. Mierzejewska J. Thomas C. M. Jagura-Burdzy G. **(**2004**).** ParB of *Pseudomonas aeruginosa*: interactions with its partner ParA and its target *parS* and specific effects on bacterial growth. J Bacteriol 186, 6983–6998. 10.1128/JB.186.20.6983-6998.2004 15466051 PMC522188

[r3] Bartosik A. A. Mierzejewska J. Thomas C. M. Jagura-Burdzy G. **(**2009**).** ParB deficiency in *Pseudomonas aeruginosa* destabilizes the partner protein ParA and affects a variety of physiological parameters. Microbiology 155, 1080–1092. 10.1099/mic.0.024661-0 19332810 PMC2895232

[r4] Bignell C. R. Haines A. S. Khare D. Thomas C. M. **(**1999**).** Effect of growth rate and *incC* mutation on symmetric plasmid distribution by the IncP-1 partitioning apparatus. Mol Microbiol 34, 205–216. 10.1046/j.1365-2958.1999.01565.x 10564465

[r5] Bingle L. E. Macartney D. P. Fantozzi A. Manzoor S. E. Thomas C. M. **(**2005**).** Flexibility in repression and cooperativity by KorB of broad host range IncP-1 plasmid RK2. J Mol Biol 349, 302–316. 10.1016/j.jmb.2005.03.062 15890197

[r6] Bowman G. R. Comolli L. R. Zhu J. Eckart M. Koenig M. Downing K. H. Moerner W. E. Earnest T. Shapiro L. **(**2008**).** A polymeric protein anchors the chromosomal origin/ParB complex at a bacterial cell pole. Cell 134, 945–955. 10.1016/j.cell.2008.07.015 18805088 PMC2745220

[r7] Breier A. M. Grossman A. D. **(**2007**).** Whole-genome analysis of the chromosome partitioning and sporulation protein Spo0J (ParB) reveals spreading and origin-distal sites on the *Bacillus subtilis* chromosome. Mol Microbiol 64, 703–718. 10.1111/j.1365-2958.2007.05690.x 17462018

[r8] Cervin M. A. Spiegelman G. B. Raether B. Ohlsen K. Perego M. Hoch J. A. **(**1998**).** A negative regulator linking chromosome segregation to developmental transcription in *Bacillus subtilis*. Mol Microbiol 29, 85–95. 10.1046/j.1365-2958.1998.00905.x 9701805

[r9] Churchward G. Belin D. Nagamine Y. **(**1984**).** A pSC101-derived plasmid which shows no sequence homology to other commonly used cloning vectors. Gene 31, 165–171. 10.1016/0378-1119(84)90207-5 6098521

[r10] Delbrück H. Ziegelin G. Lanka E. Heinemann U. **(**2002**).** An Src homology 3-like domain is responsible for dimerization of the repressor protein KorB encoded by the promiscuous IncP plasmid RP4. J Biol Chem 277, 4191–4198. 10.1074/jbc.M110103200 11711548

[r11] Donovan C. Schwaiger A. Krämer R. Bramkamp M. **(**2010**).** Subcellular localization and characterization of the ParAB system from *Corynebacterium glutamicum*. J Bacteriol 192, 3441–3451. 10.1128/JB.00214-10 20435732 PMC2897671

[r12] Ebersbach G. Briegel A. Jensen G. J. Jacobs-Wagner C. **(**2008**).** A self-associating protein critical for chromosome attachment, division, and polar organization in *Caulobacter*. Cell 134, 956–968. 10.1016/j.cell.2008.07.016 18805089 PMC2614312

[r13] El-Sayed A. K. Hothersall J. Thomas C. M. **(**2001**).** Quorum-sensing-dependent regulation of biosynthesis of the polyketide antibiotic mupirocin in *Pseudomonas fluorescens* NCIMB 10586. Microbiology 147, 2127–2139. 11495990 10.1099/00221287-147-8-2127

[r14] Figge R. M. Easter J. Jr Gober J. W. **(**2003**).** Productive interaction between the chromosome partitioning proteins, ParA and ParB, is required for the progression of the cell cycle in *Caulobacter crescentus*. Mol Microbiol 47, 1225–1237. 10.1046/j.1365-2958.2003.03367.x 12603730

[r15] Fogel M. A. Waldor M. K. **(**2006**).** A dynamic, mitotic-like mechanism for bacterial chromosome segregation. Genes Dev 20, 3269–3282. 10.1101/gad.1496506 17158745 PMC1686604

[r16] Gerdes K. Møller-Jensen J. Jensen R. B. **(**2000**).** Plasmid and chromosome partitioning: surprises from phylogeny. Mol Microbiol 37, 455–466. 10.1046/j.1365-2958.2000.01975.x 10931339

[r17] Glaser P. Sharpe M. E. Raether B. Perego M. Ohlsen K. Errington J. **(**1997**).** Dynamic, mitotic-like behavior of a bacterial protein required for accurate chromosome partitioning. Genes Dev 11, 1160–1168. 10.1101/gad.11.9.1160 9159397

[r18] Godfrin-Estevenon A.-M. Pasta F. Lane D. **(**2002**).** The *parAB* gene products of *Pseudomonas putida* exhibit partition activity in both *P. putida* and *Escherichia coli*. Mol Microbiol 43, 39–49. 10.1046/j.1365-2958.2002.02735.x 11849535

[r19] Gruber S. Errington J. **(**2009**).** Recruitment of condensin to replication origin regions by ParB/Spo0J promotes chromosome segregation in *B. subtilis*. Cell 137, 685–696. 10.1016/j.cell.2009.02.035 19450516

[r20] Irani V. R. Rowe J. J. **(**1997**).** Enhancement of transformation in *Pseudomonas aeruginosa* PAO1 by Mg^2+^ and heat. Biotechniques 22, 54–56. 8994645 10.2144/97221bm09

[r21] Ireton K. Gunther N. W. IV Grossman A. D. **(**1994**).** *spo0J* is required for normal chromosome segregation as well as the initiation of sporulation in *Bacillus subtilis*. J Bacteriol 176, 5320–5329. 8071208 10.1128/jb.176.17.5320-5329.1994 PMC196717

[r22a] Jagura-Burdzy G. Ibbotson J. P. Thomas C. M. **(**1991**).** The *korF* region of broad-host-range plasmid RK2 encodes two polypeptides with transcriptional repressor activity. J Bacteriol 173, 826–833. 1987165 10.1128/jb.173.2.826-833.1991 PMC207077

[r22] Jagura-Burdzy G. Thomas C. M. **(**1995**).** Purification of KorA protein from broad host range plasmid RK2: definition of a hierarchy of KorA operators. J Mol Biol 253, 39–50. 10.1006/jmbi.1995.0534 7473715

[r23] Jakimowicz D. Chater K. F. Zakrzewska-Czerwínska J. **(**2002**).** The ParB protein of *Streptomyces coelicolor* A3(2) recognizes a cluster of *parS* sequences within the origin-proximal region of the linear chromosome. Mol Microbiol 45, 1365–1377. 10.1046/j.1365-2958.2002.03102.x 12207703

[r24] Jakimowicz D. Mouz S. Zakrzewska-Czerwinska J. Chater K. F. **(**2006**).** Developmental control of a *parAB* promoter leads to formation of sporulation-associated ParB complexes in *Streptomyces coelicolor*. J Bacteriol 188, 1710–1720. 10.1128/JB.188.5.1710-1720.2006 16484182 PMC1426544

[r25] Jakimowicz D. Zydek P. Kois A. Zakrzewska-Czerwińska J. Chater K. F. **(**2007**).** Alignment of multiple chromosomes along helical ParA scaffolding in sporulating *Streptomyces* hyphae. Mol Microbiol 65, 625–641. 10.1111/j.1365-2958.2007.05815.x 17635186

[r26] Kadoya R. Baek J. H. Sarker A. Chattoraj D. K. **(**2011**).** Participation of chromosome segregation protein ParAI of *Vibrio cholerae* in chromosome replication. J Bacteriol 193, 1504–1514. 10.1128/JB.01067-10 21257772 PMC3067663

[r27] Kahn M. R. Kolter R. Thomas C. M. Figurski D. Meyer R. Remaut E. Helinski D. R. **(**1979**).** Plasmid cloning vehicles derived from plasmids ColE1, F, R6K, and RK2. Methods Enzymol 68, 268–280. 10.1016/0076-6879(79)68019-9 232215

[r28] Karimova G. Pidoux J. Ullmann A. Ladant D. **(**1998**).** A bacterial two-hybrid system based on a reconstituted signal transduction pathway. Proc Natl Acad Sci U S A 95, 5752–5756. 10.1073/pnas.95.10.5752 9576956 PMC20451

[r29] Katoh K. Toh H. **(**2008**).** Recent developments in the MAFFT multiple sequence alignment program. Brief Bioinform 9, 286–298. 10.1093/bib/bbn013 18372315

[r30] Khare D. Ziegelin G. Lanka E. Heinemann U. **(**2004**).** Sequence-specific DNA binding determined by contacts outside the helix-turn-helix motif of the ParB homolog KorB. Nat Struct Mol Biol 11, 656–663. 10.1038/nsmb773 15170177

[r31] Kim H.-J. Calcutt M. J. Schmidt F. J. Chater K. F. **(**2000**).** Partitioning of the linear chromosome during sporulation of *Streptomyces coelicolor* A3(2) involves an *oriC*-linked *parAB* locus. J Bacteriol 182, 1313–1320. 10.1128/JB.182.5.1313-1320.2000 10671452 PMC94417

[r32] Kovach M. E. Elzer P. H. Hill D. S. Robertson G. T. Farris M. A. Roop R. M. II Peterson K. M. **(**1995**).** Four new derivatives of the broad-host-range cloning vector pBBR1MCS, carrying different antibiotic-resistance cassettes. Gene 166, 175–176. 10.1016/0378-1119(95)00584-1 8529885

[r33] Kusiak M. Gapczynska A. Plochocka D. Thomas C. M. Jagura-Burdzy G. **(**2011**).** Binding and spreading of ParB on DNA determine its biological function in *Pseudomonas aeruginosa*. J Bacteriol 193, 3342–3355. 10.1128/JB.00328-11 21531806 PMC3133298

[r34] Larkin M. A. Blackshields G. Brown N. P. Chenna R. McGettigan P. A. McWilliam H. Valentin F. Wallace I. M. Wilm A. **& other authors (**2007**).** clustal w and clustal_x version 2.0. Bioinformatics 23, 2947–2948. 10.1093/bioinformatics/btm404 17846036

[r35] Lasocki K. Bartosik A. A. Mierzejewska J. Thomas C. M. Jagura-Burdzy G. **(**2007**).** Deletion of the *parA* (*soj*) homologue in *Pseudomonas aeruginosa* causes ParB instability and affects growth rate, chromosome segregation, and motility. J Bacteriol 189, 5762–5772. 10.1128/JB.00371-07 17545287 PMC1951838

[r36] Lee P. S. Grossman A. D. **(**2006**).** The chromosome partitioning proteins Soj (ParA) and Spo0J (ParB) contribute to accurate chromosome partitioning, separation of replicated sister origins, and regulation of replication initiation in *Bacillus subtilis*. Mol Microbiol 60, 853–869. 10.1111/j.1365-2958.2006.05140.x 16677298

[r37] Lee P. S. Lin D. C.-H. Moriya S. Grossman A. D. **(**2003**).** Effects of the chromosome partitioning protein Spo0J (ParB) on *oriC* positioning and replication initiation in *Bacillus subtilis*. J Bacteriol 185, 1326–1337. 10.1128/JB.185.4.1326-1337.2003 12562803 PMC142880

[r38] Leonard T. A. Butler P. J. Löwe J. **(**2004**).** Structural analysis of the chromosome segregation protein Spo0J from *Thermus thermophilus*. Mol Microbiol 53, 419–432. 10.1111/j.1365-2958.2004.04133.x 15228524

[r39] Lewis R. A. Bignell C. R. Zeng W. Jones A. C. Thomas C. M. **(**2002**).** Chromosome loss from *par* mutants of *Pseudomonas putida* depends on growth medium and phase of growth. Microbiology 148, 537–548. 11832517 10.1099/00221287-148-2-537

[r40] Lin D. C.-H. Grossman A. D. **(**1998**).** Identification and characterization of a bacterial chromosome partitioning site. Cell 92, 675–685. 10.1016/S0092-8674(00)81135-6 9506522

[r41] Marston A. L. Errington J. **(**1999**).** Dynamic movement of the ParA-like Soj protein of *B. subtilis* and its dual role in nucleoid organization and developmental regulation. Mol Cell 4, 673–682. 10.1016/S1097-2765(00)80378-0 10619015

[r42] Mohl D. A. Gober J. W. **(**1997**).** Cell cycle-dependent polar localization of chromosome partitioning proteins in *Caulobacter crescentus*. Cell 88, 675–684. 10.1016/S0092-8674(00)81910-8 9054507

[r43] Mohl D. A. Easter J. Jr Gober J. W. **(**2001**).** The chromosome partitioning protein, ParB, is required for cytokinesis in *Caulobacter crescentus*. Mol Microbiol 42, 741–755. 10.1046/j.1365-2958.2001.02643.x 11722739

[r44] Mullis K. Faloona F. Scharf S. Saiki R. Horn G. Erlich H. **(**1986**).** Specific enzymatic amplification of DNA *in vitro*: the polymerase chain reaction. Cold Spring Harb Symp Quant Biol 51, 263–273. 10.1101/SQB.1986.051.01.032 3472723

[r45] Murray H. Errington J. **(**2008**).** Dynamic control of the DNA replication initiation protein DnaA by Soj/ParA. Cell 135, 74–84. 10.1016/j.cell.2008.07.044 18854156

[r46] Notredame C. Higgins D. G. Heringa J. **(**2000**).** T-Coffee: a novel method for fast and accurate multiple sequence alignment. J Mol Biol 302, 205–217. 10.1006/jmbi.2000.4042 10964570

[r47] Ogura Y. Ogasawara N. Harry E. J. Moriya S. **(**2003**).** Increasing the ratio of Soj to Spo0J promotes replication initiation in *Bacillus subtilis*. J Bacteriol 185, 6316–6324. 10.1128/JB.185.21.6316-6324.2003 14563866 PMC219394

[r48] Ptacin J. L. Lee S. F. Garner E. C. Toro E. Eckart M. Comolli L. R. Moerner W. E. Shapiro L. **(**2010**).** A spindle-like apparatus guides bacterial chromosome segregation. Nat Cell Biol 12, 791–798. 10.1038/ncb2083 20657594 PMC3205914

[r49] Quisel J. D. Grossman A. D. **(**2000**).** Control of sporulation gene expression in *Bacillus subtilis* by the chromosome partitioning proteins Soj (ParA) and Spo0J (ParB). J Bacteriol 182, 3446–3451. 10.1128/JB.182.12.3446-3451.2000 10852876 PMC101922

[r50] Quisel J. D. Lin D. C. Grossman A. D. **(**1999**).** Control of development by altered localization of a transcription factor in *B. subtilis*. Mol Cell 4, 665–672. 10.1016/S1097-2765(00)80377-9 10619014

[r51] Rashid M. H. Kornberg A. **(**2000**).** Inorganic polyphosphate is needed for swimming, swarming, and twitching motilities of *Pseudomonas aeruginosa*. Proc Natl Acad Sci U S A 97, 4885–4890. 10.1073/pnas.060030097 10758151 PMC18327

[r52] Real G. Autret S. Harry E. J. Errington J. Henriques A. O. **(**2005**).** Cell division protein DivIB influences the Spo0J/Soj system of chromosome segregation in *Bacillus subtilis*. Mol Microbiol 55, 349–367. 10.1111/j.1365-2958.2004.04399.x 15659156

[r53] Rodionov O. Yarmolinsky M. **(**2004**).** Plasmid partitioning and the spreading of P1 partition protein ParB. Mol Microbiol 52, 1215–1223. 10.1111/j.1365-2958.2004.04055.x 15130136

[r54] Rodionov O. Lobocka M. Yarmolinsky M. **(**1999**).** Silencing of genes flanking the P1 plasmid centromere. Science 283, 546–549. 10.1126/science.283.5401.546 9915704

[r55] Saint-Dic D. Frushour B. P. Kehrl J. H. Kahng L. S. **(**2006**).** A *parA* homolog selectively influences positioning of the large chromosome origin in *Vibrio cholerae*. J Bacteriol 188, 5626–5631. 10.1128/JB.00250-06 16855253 PMC1540020

[r56] Sambrook J. Fritsch E. F. Maniatis T. **(**1989**).** Molecular Cloning: a Laboratory Manual, 2nd edn Cold Spring Harbor, NY: Cold Spring Harbor Laboratory.

[r57] Schofield W. B. Lim H. Ch. Jacobs-Wagner C. **(**2010**).** Cell cycle coordination and regulation of bacterial chromosome segregation dynamics by polarly localized proteins. EMBO J 29, 3068–3081. 10.1038/emboj.2010.207 20802464 PMC2944072

[r58] Scholefield G. Whiting R. Errington J. Murray H. **(**2011**).** Spo0J regulates the oligomeric state of Soj to trigger its switch from an activator to an inhibitor of DNA replication initiation. Mol Microbiol 79, 1089–1100. 10.1111/j.1365-2958.2010.07507.x 21235642

[r59] Schumacher M. A. Mansoor A. Funnell B. E. **(**2007**).** Structure of a four-way bridged ParB-DNA complex provides insight into P1 segrosome assembly. J Biol Chem 282, 10456–10464. 10.1074/jbc.M610603200 17293348

[r60] Schumacher M. A. Piro K. M. Xu W. **(**2010**).** Insight into F plasmid DNA segregation revealed by structures of SopB and SopB-DNA complexes. Nucleic Acids Res 38, 4514–4526. 10.1093/nar/gkq161 20236989 PMC2910045

[r61] Sharpe M. E. Errington J. **(**1996**).** The *Bacillus subtilis soj-spo0J* locus is required for a centromere-like function involved in prespore chromosome partitioning. Mol Microbiol 21, 501–509. 10.1111/j.1365-2958.1996.tb02559.x 8866474

[r62] Simon R. O’Connell M. Labes M. Pühler A. **(**1986**).** Plasmid vectors for the genetic analysis and manipulation of rhizobia and other Gram-negative bacteria. Methods Enzymol 118, 640–659. 10.1016/0076-6879(86)18106-7 3005803

[r63] Sullivan N. L. Marquis K. A. Rudner D. Z. **(**2009**).** Recruitment of SMC by ParB–*parS* organizes the origin region and promotes efficient chromosome segregation. Cell 137, 697–707. 10.1016/j.cell.2009.04.044 19450517 PMC2892783

[r64] Thanbichler M. Shapiro L. **(**2006**).** MipZ, a spatial regulator coordinating chromosome segregation with cell division in *Caulobacter*. Cell 126, 147–162. 10.1016/j.cell.2006.05.038 16839883

[r65] Toro E. Hong S.-H. McAdams H. H. Shapiro L. **(**2008**).** *Caulobacter* requires a dedicated mechanism to initiate chromosome segregation. Proc Natl Acad Sci U S A 105, 15435–15440. 10.1073/pnas.0807448105 18824683 PMC2563096

[r66] Viollier P. H. Thanbichler M. McGrath P. T. West L. Meewan M. McAdams H. H. Shapiro L. **(**2004**).** Rapid and sequential movement of individual chromosomal loci to specific subcellular locations during bacterial DNA replication. Proc Natl Acad Sci U S A 101, 9257–9262. 10.1073/pnas.0402606101 15178755 PMC438963

[r67] Webb C. D. Teleman A. Gordon S. Straight A. Belmont A. Lin D. C.-H. Grossman A. D. Wright A. Losick R. **(**1997**).** Bipolar localization of the replication origin regions of chromosomes in vegetative and sporulating cells of *B. subtilis*. Cell 88, 667–674. 10.1016/S0092-8674(00)81909-1 9054506

[r68] Williams D. R. Thomas C. M. **(**1992**).** Active partitioning of bacterial plasmids. J Gen Microbiol 138, 1–16. 1556544 10.1099/00221287-138-1-1

[r69] Yamaichi Y. Niki H. **(**2000**).** Active segregation by the *Bacillus subtilis* partitioning system in *Escherichia coli*. Proc Natl Acad Sci U S A 97, 14656–14661. 10.1073/pnas.97.26.14656 11121066 PMC18974

